# Effects of Replacing Inorganic with Organic Glycinates Trace Minerals on Growth Performance, Gut Function, and Minerals Loss of Juvenile Nile Tilapia

**DOI:** 10.1155/2024/1422124

**Published:** 2024-06-27

**Authors:** Allan Vinnicius Urbich, Thais Pereira da Cruz, Bruno Wernick, Ibrahim Adeshina, Aline Victoria Sampaio, Giovani Sampaio Gonçalves, Adriana Yuriko Koga Kozu, Leandro Cavalcante Lipinski, Valéria Rossetto Barriviera Furuya, Wilson Massamitu Furuya

**Affiliations:** ^1^ Animal Science Graduate Degree Program State University of Maringá, Maringá, PR, Brazil; ^2^ BASF Animal Nutrition, São Paulo, SP, Brazil; ^3^ Department of Aquaculture and Fisheries University of Ilorin, Ilorin, Nigeria; ^4^ College Animal Science State University of Ponta Grossa, Ponta Grossa, PR, Brazil; ^5^ São Paulo Agribusiness Technology Agency—APTA, São José do Rio Preto, São Paulo, Brazil; ^6^ Department of Medicine State University of Ponta Grossa, Ponta Grosa, PR, Brazil; ^7^ Department of Animal Science State University of Ponta Grossa, Ponta Grosa, PR, Brazil

## Abstract

This study aimed to investigate the effects of replacing inorganic trace minerals (ITM) with organic trace minerals (OTM) on growth performance, body composition, gut histomorphometry, digestive enzyme activity, apparent digestibility coefficients (ADC), and mineral balance in juvenile Nile tilapia, *Oreochromis niloticus*. Fish (*n* = 432; 2.5 ± 0.5 g) were randomly distributed into four groups with four replicates each (27 fish per replicate). A control diet (gross 15.2 MJ gross energy kg^−1^; 367.3 g crude protein kg^−1^) was elaborated to meet the dietary requirements of zinc (Zn), manganese (Mn), and copper (Cu) using ITM in the sulfate form (ITM100). From the control diet, three diets were elaborated using OTM to supply 100% (OTM100), 75% (OTM75), and 50% (OTM50) of Zn, Mn, and Cu ITM of the control diet, respectively, supplied in the glycinate form. Fish were hand-fed six times daily for 8 weeks. Growth performance was not changed by dietary treatments. Fish fed on the diet OTM50 showed higher whole-body and vertebrae ash contents and higher whole-body calcium (Ca), Zn, and iron (Fe) retention than those fed on the control diet. The activity of digestive enzymes and the ADC of energy and nutrients, including Zn, Mn, and Cu, were similar in fish fed on diets OTM50 and ITM100. A reduction in Zn (−39.8%), Mn (−11.1%), and Cu (−14.0%) loss was observed in fish fed on the diet OTM50 than in fish fed diet ITM100. The dietary treatments did not affect the gut histomorphometry. In conclusion, the inclusion of OTM in Nile tilapia's diets represents a promising strategy to reduce 50% of ITM sources by utilizing Zn, Mn, and Cu OTM as glycinates without detrimental effects on growth performance, nutrient utilization, and gut function. These results additionally reinforce the environmental benefits of using OTM for precise mineral nutrition in aquaculture.

## 1. Introduction

Nile tilapia, *Oreochromis niloticus*, is widely known as a promising fish species that is widely produced because of its rapid growth and remarkable market acceptance [1, 2]. It accounted for 8% of the world's total finfish production, representing the fourth-largest finfish species (4.2 mt) [3]. Therefore, implementing precise nutritional farming is a critical strategy for expanding tilapia production, thereby enhancing productivity and profitability [4]. Recently, mineral nutrition has been highlighted to input the precise nutrition concept, considering the growth, health, and sustainability principles of several fish species [5].

Trace minerals (TM) are an essential component in the diet of all animals, including fish, because they play a crucial role in skeletal development, embryonic development, colloidal system maintenance, regulation of acid–base equilibrium, and biologically important compounds like hormones and enzymes, as well as other essential biochemical processes [6, 7]. Notably, copper (Cu), a trace element, is an essential part of numerous enzyme systems and acts as a cofactor for the enzymes cytochrome oxidase and superoxide dismutase [8]. Additionally, it is essential for controlling lysyl oxidase activity [9], and its deficiency may result in malabsorption syndrome by damaging the connective tissues in the digestive system [10]. Similarly, manganese (Mn) is a critical part of metalloenzymes and is involved in the metabolism of amino acids, fatty acids, and glucose [11]. Getting enough Mn is essential for creating cellular energy, which is necessary to operate all body systems properly. Ultimately, as a cofactor for several enzymes and hormones, zinc (Zn) plays a role in animal immunomodulation [12].

Due to affordability and availability at a commercial level, TM are typically supplemented in the diet due to their deficiency in feed ingredients [13]. They are mainly produced from inorganic compounds like sulfates, oxides, chlorides, and phosphates [5]. Inorganic trace minerals (ITM) are unstable, rapidly dissociate in the digestive tract, and interact with other substances, which causes them to be lost before absorption [14]. Consequently, higher dietary ITM levels are required to meet fish requirements [15, 16], which ultimately raises production costs and causes environmental issues. ITM could be replaced with organic trace minerals (OTM) to solve these issues. OTM are chelated or complexed minerals with organic compounds such as amino acids, proteins, or organic acids. OTM are more stable due to their organically bound structure with better digestion and absorption in the intestine [17], which, in turn, increase their bioavailability and assimilability [18] and consequently reduce fecal and urinary excretion [19].

Due to their excellent stability, palatability, and electrical neutrality, glycinate minerals are the preferred organic source of TM [20]. The stability of chelate complexes is favored by glycine, the amino acid with the lowest molecular weight, and prevents TM release in the stomach and intestine [21]. The glycinate-TM forms of iron (Fe; Fe-Gly) and Zn (Zn-Gly) have received the most research attention. Similarly, Zn-Gly outperformed Zn sulfate (ZnSO_4_) in performance [22]. Zn-Gly enhances intestinal Zn absorption, which is positively correlated with broiler growth performance [22, 23]. At the same dose rate, Zn-Gly demonstrated considerably higher Zn retention in the muscle, liver, and albumen than ZnSO_4_ fed to broiler breeders [20]. Additionally, previous study has evidenced that Zn-Gly supplementation dramatically increased intestinal maturity, which is associated with higher fish growth performance [22].

According to various studies, organically complexed or chelated TM are more effective and stable than their inorganic counterparts. An improved growth was reported in rainbow trout, *Oncorhynchus mykiss*, due to the supplementation of amino acid-chelated trace elements in practical diets for this fish species has been documented [24, 25]. Partial ITM replacement with OTM did not significantly improve final growth and feed utilization, but phosphorus (P) absorption was augmented with the addition of OTM; bone and liver mineral contents were higher in the fish-fed OTM than in the ITM group [26]. Similarly, hematocrit level and alkaline phosphates (ALP) activity were highly elevated in the chelate-fed fish [27]. However, limited research has explored the effects of substituting ITM with OTM in Nile tilapia concerning the outputs of Zn, Mn, and Cu.

Heavy metal accumulation in aquaculture farms has become a significant issue due to the use of artificial feed to boost fish productivity [28]. Remarkably, recent studies have evidenced Zn, Mn, and Cu as heavy metal contaminations in the water of aquaculture farms in tropical areas [29]. However, there is currently a gap in our knowledge regarding using ITM versus OTM to enhance the sustainability of fish production. Therefore, the current study aims to determine how replacing 100%, 75%, or 50% Zn, Mn, and Cu ITM by OTM affects juvenile Nile tilapia's growth performance, energy and nutrient utilization, gut histomorphometry, and mineral balance.

## 2. Materials and Methods

### 2.1. Ethics Statement

The Animal Care and Use Committee of the State University of Ponta Grossa approved the current experiment protocol (Permit number 24302-4). Anesthesia procedures were conducted using tricaine methanesulfonate (MS-222) (Sigma-Aldrich, São Paulo, SP, Brazil) at 200 mg L^−1^ [30]. Euthanasia was carried out following the two-step procedure of the American Veterinary Medical Association [31] via immersion in an MS-222 solution (1,000 mg L^−1^ water) and the secondary penetrating captive bolt method.

### 2.2. Diets

A control diet was elaborated to meet the dietary requirements of protein (367.3 g kg^−1^), energy (15.2 MJ kg^−1^), and other nutrients, including Zn, Mn, and Cu from inorganic sulfate minerals to meet the dietary requirement (ITM100) for Nile tilapia [32]. From the control diet, three diets were elaborated using organic Zn-glycinate, Mn-glycinate, and Cu-glycinate (BASF, Corporation, Ludwigshafen, Germany) to provide the respective minerals at 100% (OTM100), 75% (OTM75), and 50% (OTM50) of the dietary requirements established in the control diet (Tables 1 and 2). The control diet was formulated based on the food composition, which was confirmed by laboratory analysis after the extrusion and drying procedures of the diets. The dietary requirements were established at 79.5 mg kg^−1^ of Zn [33], 7 mg kg^−1^ of Mn [34], and 4 mg kg^−1^ of Cu [35] for the control diet used in the present study. Feed ingredients were mixed in a vertical mixer (Incomagri 1205, Itapira, SP, Brazil) and ground in a centrifugal mill (Viera MC 680B, Tatuí, SP, Brazil) using a sieve featuring 0.8 mm diameter holes. The extrusion process was performed in a single-screw laboratory extruder (Exteec EX30, Ribeirão Preto, SP, Brazil) utilizing a die hole size of 1.5 mm in diameter, three knives rotating at 40 rpm, and an end die temperature of 92°C producing pellets with a diameter of 2.0–2.2 mm. The diets were then dried in a forced-air ventilation oven (Tecnal TE-394/2-MP, Piracicaba, SP, Brazil) at 55°C until a uniform moisture level of 7% was achieved (−24 hr).

### 2.3. Fish, Experimental Conditions, and Experimental Desing

A total of 1,000 masculinized Nile tilapia fry with an initial weight of 1.0 ± 0.1 g (Genomar, AquaGenetics do Brasil Ltda, Rolândia, PR, Brazil) were acclimated for a 4-week period in two circular fiberglass aquariums of 200 L each. The temperature and dissolved oxygen were maintained at 28 ± 0.5°C and 6.2 ± 0.2 mg L^−1^, respectively. During the acclimatization period, fish were hand-fed on a commercial extruded diet (Supra-Alisul Alimentos 1.5 mm diameter, Maringá, PR, Brazil) containing analyzed 452 g kg^−1^ of crude protein, 12 times daily until apparent satiety. For the experimental phase, out of the 1,000 fish acclimated, 432 individuals (mean weight: 2.5 ± 0.5 g; mean ± SD) displaying a body weight gain (BWG) within ± 30% of the population mean were randomly distributed into 16 aquaria of 100 L each. The experimental design followed a completely randomized arrangement to assess four distinct diets, with each replicate aquarium containing 27 fish. The aquaria were part of a recirculating aquaculture system that included a decanter for solid removal, a mechanical filter with bio-balls (32 mm diameter each), a 3,000 W heater, and a UV-light disinfection system with a 55 W rating. Aeration was provided by a 0.5-HP centrifugal blower (Sulpesca, Toledo, PR, Brazil) connected to each aquarium through silicone airline tubing and porous stones. The water temperature was maintained at 28 ± 0.2°C, and dissolved oxygen levels were kept at 6.2 ± 0.2 mg L^−1^ throughout the feeding trial. Temperature and dissolved oxygen data were monitored daily in each aquarium using a multiparameter oximeter (YSI Incorporated, Ohio, USA). The trial followed a natural photoperiod of 12 hr light and 12 hr dark. Water quality parameters were monitored weekly and recorded as follows (mean ± SD): pH (6.8 ± 0.23), total ammonia (0.01 ± 0.007 mg L^−1^), nitrite (0.02 ± 0.01 mg L^−1^), and nitrate (0.01 ± 0.005 mg L^−1^). pH measurements were obtained using a digital pH meter (TEC-2, Piracicaba, SP, Brazil). Additionally, ammonia, nitrite, and nitrate levels were analyzed using commercial kits (Alfakit, Florianópolis, SC, Brazil).

### 2.4. Sample Collection

After acclimation, 90 fish were euthanized and randomly sampled for initial whole-body composition determination. After 8 weeks of the experimental trial, all fish underwent a 24-hr fasting period, followed by anesthesia, counting, and group-weighing. Subsequently, seven fish from each aquarium were randomly selected, euthanized, and sampled for doing the whole-body composition, while an additional eight fish were collected for vertebrae mineral deposition analysis. Fish samples for whole-body composition analysis were immediately ground using a meat grinder, subsequently dried in a ventilated oven at 55°C (Tecnal, TE-394/1-MP, Piracicaba, SP, Brazil) for 72 hr, and stored at −20°C until analysis. Concurrently, three additional fish from each aquarium were randomly selected and anesthetized for blood analysis. Pooled blood (2 mL aliquots) was collected from the caudal vein using a 1-mL syringe fitted with a 0.45 × 13 mm needle for subsequent biochemical analysis. The blood samples were immediately centrifuged (Kasvi K14-0802, Pinhais, PR, Brazil) at 3,500 × *g* for 10 min at 4°C to achieve plasma, which was kept in 2-mL cryostat tube and stored at −80°C until analysis. Furthermore, the same fish collected for blood parameters were utilized to measure the hepatosomatic index, visceral fat content, relative intestine length, and digestive enzyme activity, in which the liver and visceral fat were removed on ice. Gut fragments were then collected to evaluate digestive enzyme activity using a well-established method with slight modifications [36, 37]. Specifically, from the same fish used for blood collection analysis, a 4 cm section of the middle section of the intestine, located 40 cm distal to the stomach-intestine junction, was sampled and opened longitudinally. The fragment was then washed with standard saline solution to remove any remaining intestinal content before being stored at −80°C until the analysis of digestive enzyme activity. Additionally, a 1 cm section of the middle intestine was collected from the same fish and fixed in buffered formalin (10%) for 24 hr for histological analysis.

### 2.5. Chemical Composition

The chemical composition of feed ingredients, diets, feces, and whole-body samples was determined for dry matter, crude protein, crude lipid, ash [38], and crude fiber [39]. Dry matter was analyzed by drying the samples overnight in an oven at 105°C. For the determination of crude protein (N × 6.25), a micro-Kjeldahl apparatus (Tecnal, MA-036, Piracicaba, SP, Brazil) was employed, while crude lipid content was determined through ether extraction using a Tecnal TE-044 apparatus (Piracicaba, SP, Brazil). Crude fiber analysis involved a sequential process, comprising digestion with a sulfuric acid solution followed by a sodium hydroxide solution, leading to the complete combustion of organic matter at 600°C for 4 hr. The mineral content was determined by incinerating the sample in a muffle furnace (Belo Horizonte B200, MG, Brazil) at 550°C for 6 hr. For the determination of calcium (Ca), P, Zn, magnesium, Mn, Fe, Cu, and chromium (Cr), inductively coupled plasma optical emissions spectrometry (PerkinElmer 8000, Waltham, MA, USA) was employed. The analysis utilized an internally validated method [40]. For the determination of both macro and micro minerals, closed-vessel microwave-assisted digestion was conducted utilizing a PerkinElmer MPS 320™ microwave digestion system. The analysis of gross energy in feed ingredients, diets, and feces was determined using an adiabatic bomb calorimeter (Parr6400; Parr Instruments Co., Moline, IL, USA) using benzoic acid as the calibration standard. All samples were analyzed in duplicate.

### 2.6. Calculation

Growth performance was assessed using the following metrics: Weight gain (%) = 100 × [(final weight (g) − initial weight (g))/(initial weight (g))]; feed intake (%) of body weight per day (% BW day^−1^) = 100 × [dry feed intake (g) average fish weight (g)/days fed]; feed efficiency ratio = [weight gain (g)/dry feed consumed (g)]; protein retention efficiency (%) = 100 × [protein gain (g)/protein intake (g)]; energy retention efficiency, % = 100 × [energy gain (g)/energy intake (g)]; viscerosomatic index (%) = 100 × [(visceral weight (g)/body weight (g)]; hepatosomatic index (%) = 100 × [liver weight (g)/body weight (g)]; visceral fat (%) = 100 × [visceral fat weight (g)/visceral fat (g)]; survival (%) = 100 × [(final number of fish/initial number of fish)].

### 2.7. Blood Parameters

Blood parameters were determined using spectrometry with a semiautomatic biochemical analyzer (BIO-2000 IL, Barueri, SP, Brazil) and commercial kits from BIOTÉCNICA® (Varginha, MG, Brazil). The parameters analyzed included total protein (Catalog 90.019.00), triglycerides (Catalog 90.022.00), total cholesterol (Catalog 90.021.00), and glucose (Catalog 90.017.00) content. Additionally, the activity of alanine transaminase (ALT; Catalog 90.013.00), aspartate aminotransferase (AST; Catalog 90.014.00), and ALP (Catalog 90.015.00) enzymes was determined.

### 2.8. Activity of Digestive Enzymes

The activity of the digestive enzymes of *α*-amylase, alkaline protease, and lipase was assessed using previously established methods [41]. Alkaline protease activity analysis was assessed using Azocasein (2%) (Sigma–Aldrich, São Paulo, SP, Brazil) as the substrate in a Tris–HCl buffer with pH = 7.5 (Sigma–Aldrich, São Paulo, SP, Brazil). Enzyme activity was quantified as 1 *μ*M of *n*-nitrophenol released per mg protein per minute. Lipase-specific activity was evaluated using nitrophenyl myristate (Sigma–Aldrich, São Paulo, SP, Brazil) as the substrate. Each assay (0.5 mL) contained 0.53 mM p-nitrophenyl myristate, 0.25 mM 2-methoxy ethanol, 5 mM sodium cholate, and 0.25 M Tris–HCl buffer (pH = 9.0). After incubation for 15 min (30°C), the reaction was ended by adding 0.7 mL of acetone/*n*-heptane (5 : 2, v/v). Following centrifugation at 6,080 × *g* for 2 min., the optical density of the aqueous solution was measured (405 nm), and the enzyme activity was expressed as 1 *μ*M of *n*-nitrophenol released per mg protein per min. *α*-Amylase activity was assessed using starch as the substrate [42]. In brief, the crude enzyme extract was incubated with starch solution (1% w/v) in a 0.02 M sodium phosphate buffer containing 0.006 M NaCl (pH = 6.9) for 4 min. at 25°C. After incubation, 0.5 mL of dinitrosalicylic acid (Sigma–Aldrich, São Paulo, SP, Brazil) solution (1% w/v) was added, and the mixture was boiled 5 min and cooled to room temperature. The optical density was measured after adding 5 mL of distilled water, and *α*-amylase activity was expressed as *μ*M of maltose produced per mg protein per minute at 25°C.

### 2.9. Digestibility Assay

The apparent digestibility coefficients (ADC) of gross energy and nutrients were determined using chromic oxide (Cr_2_O_3_) as an external inert marker [43]. The fecal collection followed a previously established methodology with slight modifications [44]. During the 1-month feeding trial, feces were collected daily from each aquarium until the day before the end of the experiment. At 8:00 p.m., the bottom of each aquarium was thoroughly cleaned, and fish were given a 2-hr feeding period before feces collection at 10:00 p.m. The feces were manually siphoned and strained through a 1 mm mesh net. The collected fecal samples were then centrifuged at 3,500 × *g* for 10 min at room temperature. After discarding the supernatant, the sediment was dried in a ventilated oven (Tecnal TE-394/5-MP, Piracicaba, SP, Brazil) at 55°C for 24 hr. The dried feces were ground into a fine powder (0.5 mm diameter) using a laboratory Willye mill (Tecnal R-TE 648, Piracicaba, SP, Brazil) and maintained at 5°C until laboratory analysis. The ADC was determined following a previously established equation [45] as ADC = 1−[(*C*_d_/*C*_f_) × (*N*_f_/*N*_d_)], where ADC is the apparent digestibility coefficients; *C*_d_ is the concentration of chromium oxide in the diet; *C*_f_ is the concentration of chromium oxide in the feces (g kg^−1^); *N*_f_ is the concentration of energy or nutrient in the feces (g kg^−1^ or MJ kg^−1^); and *N*_d_ is the concentration of energy or nutrient in the diet (g kg^−1^ or MJ kg^−1^).

### 2.10. Gut Histomorphometry

Intestinal fragments were embedded in paraffin blocks [46]. Semi-serial 5 *µ*m cross-sections were prepared and subsequently stained with hematoxylin–eosin (HE) [47]. Villous height was measured by examining 100 intact villi per fish, resulting in 1,600 measurements per treatment. Gut histological sections were examined using an optical microscope with a Pro-Series camera attachment (Media Cybertechniques, Olympus, Japan) to capture images. Total villus height (TVH), villus width (VW), and villus epithelium thickness (VET) were measured using Image-Pro Plus software (version 5.2—CyberMedia). The area of absorption (AA) was calculated as the product of TVH and VW (TVM × VW) [48].

### 2.11. Vertebrae Minerals

Trunk and caudal vertebrae were obtained using a modified version of the previously established protocol [49]. First, fish samples were cooked in a microwave for 6 min until the flesh and bone were swiftly separated. Second, soft tissues were meticulously excised from the vertebrae. Third, the collected vertebrae bones underwent an initial rinse with distilled water, followed by drying the bone samples in an oven at 105°C for 24 hr and subsequent grinding in a laboratory hammer mill. Fourth, the vertebrae samples were defatted using a 1 : 1 chloroform–methanol solution [50]. Following the defatting procedure, the samples were air-dried for 24 hr prior to mineral analysis.

### 2.12. Minerals Balance

The Zn, Mn, and Cu balances were calculated by modifying the method previously established in fish [51, 52, 53, 54] as *M*_i_ = FI (kg kg^−1^ BWG) × *M*_f_, where *M*_i_ is mineral intake (mg kg^−1^ BWG), FI is feed intake (kg kg^−1^ BWG), and *M*_f_ is mineral in feed (mg kg^−1^); *M*_r_ = (BW_1_ × *M*_1_–BW_0_ × *P*_0_)/*M*_i_ × 100, where *M*_r_ is mineral retained (%), BW_1_ and BW_0_ are final and initial fish body weight (g), and *M*_1_ and *M*_0_ final and initial body mineral content (mg), respectively; *M*_ret_ = *M*_i_ × *M*_r_, where, *M*_ret_ is mineral retained (g kg^−1^ BWG); *M*_l_ = *M*_i_–*M*_ret_, where, *M*_l_ = mineral loss (g kg^−1^ BWG).

### 2.13. Statistical Analysis

All data are presented as the mean and standard error of the means within each treatment. Prior to analysis, the normal distribution of data was assessed using the Kolmogorov–Smirnov test. This test showed that all data followed the normal distribution pattern. The homogeneity of the residual variances was assessed and addressed through the application of Levene's test, and outlier data were subsequently treated accordingly. Statistical analyses were assessed using one-way ANOVA, and Tukey's test was applied to detect significant differences. Statistical significance was established as *P* ≤ 0.05, while 0.05 < *P* ≤ 0.10 was considered a tendency. The analysis was performed using Minitab version 19 (Minitab, Inc., State College, PA, USA).

## 3. Results

### 3.1. Growth Performance

Growth and performance were not significantly affected by the replacement of ITM with OTM in the diets of Nile tilapia (*P* ≤ 0.05; Table 3).

### 3.2. Whole-Body Proximate Composition

Table 4 depicts the whole-body composition of Nile tilapia-fed ITM100 and OTM-based diets. Whole-body moisture (*P*=0.727) and crude protein (*P*=0.401) were not affected by the replacement of ITM with OTM, while whole-body crude lipids and mineral matter were significantly (*P* ≤ 0.001) influenced by the replacement. More specifically, the fish-fed OTM100 diet had significantly lower (*P* < 0.001) whole-body crude lipids compared to the control (ITM100) and the other test (OTM75 and OTM50) diets. On the contrary, whole body mineral matter was significantly higher (*P* < 0.01) in fish-fed OM100 and OM75 diets compared to ITM100, but there was no difference between the mineral matter of fish-fed ITM100 and OTM50 diets.

### 3.3. Whole-Body and Vertebrae Minerals Composition

The whole-body and vertebrae of the fish were not significantly affected due to the replacement of ITM with OTM in Nile tilapia (*P* ≤ 0.05), as shown in Table 5. However, a tendency for higher vertebrae *P* (*P*=0.068) was found for fish-fed diet OTM75 compared to that fed other diets.

### 3.4. Whole-Body Minerals Retention

The substitution of ITM by OTM in diets of Nile tilapia did not affect the whole-body *P* (*P*=0.694), Mn (*P*=0.075), and Cu (*P*=0.138) retention. However, fish fed with the OTM50 diet displayed higher whole-body Ca (*P*=0.011), Zn (*P* < 0.001), and Fe (*P*=0.002) than those fed diet ITM100 (Table 6). Additionally, a tendency for higher whole-body Mn retention (*P*=0.075) was found for fish fed diets OTM75 and OTM50 compared to those fed diets ITM100 and OTM100.

### 3.5. Plasmatic Biochemical Profiles

Table 7 depicts the plasma biochemistry of Nile tilapia fed on OTM in the replacement of ITM diets. Interestingly, fish fed on the OTM50 diet recorded higher ALP values (*P*=0.005) relative to fish fed on the OTM100 diet. Furthermore, the activity of AST (*P*=0.212), ALT (*P*=0.080), as well as the levels of glucose (*P*=0.056), total protein (*P*=0.929), triglycerides (*P*=0.184), and cholesterol (*P*=0.720) showed no statistically significant differences among fish fed with ITM and OTM-contained diets. Notably, fish-fed diets OTM100 and OTM75 showed a tendency for higher plasmatic activity of ALP enzyme (*P*=0.080) and glucose level (*P*=0.056) compared to fish fed diet ITM100.

### 3.6. Gut Histomorphometry

The inclusion of OTM levels in Nile tilapia diets did not significantly alter villus height (*P*=0.249), villus width (*P*=0.537), villus height-villus width ratio (*P*=0.167) and area of intestinal absorption (*P*=0.237), as displayed in Table 8.

### 3.7. Digestive Enzyme Activities

**Figure 1:**
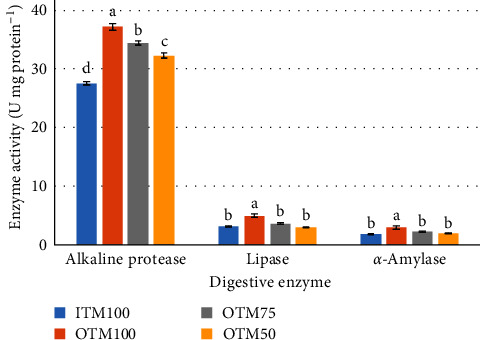
Gut digestive enzyme activity (U mg protein^−1^) of juvenile Nile tilapia fed the experimental diets over an 8-week feeding trial. *Note:* ITM100: control diet was elaborated to meet the dietary requirements of Zn, Mn, and Cu from inorganic sulfate minerals (ITM100). OTM100, OTM75, and OTM50: diets were elaborated using organic Zn-glycinate, Mn-glycinate, and Cu-glycinate to provide the respective minerals at 100% (OTM100), 75% (OTM75), and 50% (OTM50) of the dietary requirements established in the control diet, respectively. Data are means ± standard error of the mean of four replicates (27 fish per replicate). ^a–d^Bars with different superscript letters indicate significant differences for each enzyme by Tukey's test (*P* ≤ 0.05).

The protease activity was notably higher in fish fed on the OTM50 diet than those fed on other diets (*P* ≤ 0.05). Fish fed on OTM75 and OTM50 diets presented higher activity of *α*-amylase) and lipase relative to those fed on the ITM100 diet (*P* ≤ 0.05), as displayed in Figure 1.

### 3.8. ADC of Experimental Diets and Minerals Balance in Fish Body

The ADC (%) of the experimental diets offered to Nile tilapia juveniles are presented in Table 9. Fish fed on the OTM50 diet presented higher ADC of gross energy (*P* < 0.001) than those fed with other diets. The ADC of crude protein was higher (*P* < 0.001) in fish fed on OTM100 and OTM75 diets than those fed on ITM100 and OTM50 diets. Differently, the ADC of Fe was higher (*P*=0.038) in fish fed on the OTM50 diet relative to those fed on the ITM100 diet, while the ADC of Zn was higher (*P*=0.023) in fish fed on the OTM50 diet relative to those fed on the ITM100 diet. Nevertheless, the ADC of Ca (*P*=0.333), *P* (*P*=0.618), Mn (*P*=0.099), and Cu (*P*=0.201) were not affected by dietary treatments. A tendency for higher ADC of Mn (*P*=0.099) was obtained for fish-fed diets OTM100 relative to fish-fed diets OTM75 and OTM50.

Data on Zn, Mn, and Cu balance in Nile tilapia fed on the experimental diets are presented in Table 10. Markedly, fish fed on the OTM50 showed lower intake and loss of Zn (*P* < 0.001), Mn (*P* < 0.001), and Cu (*P* < 0.001) than those fed on other diets. Fish fed with the OTM100 diet retained higher Cu (*P* < 0.001) than those fed on other diets.

## 4. Discussion

This study showed no effects on Nile tilapia growth performance when substituting 100% ITM for 50% OTM to meet the dietary requirements for Zn, Mn, and Cu. Similarly, a previous study reported that OTM had no impact on Nile tilapia growth [50], and this also aligns with previous findings reported in rainbow trout [25, 26, 27]. However, the hypothesis was confirmed that OTM are more stable and possesses higher bioavailability than ITM [18, 19]. This enhanced stability and bioavailability of OTMs likely account for the similar growth performance responses observed when substituting lower levels of OTMs for ITMs in the current study.

This study found that feeding Nile tilapia OTM significantly changed the fish's body composition, particularly in crude lipid content. One possible reason is that the higher availability of OTM-Zn may promoted its excess in fish fed with the ITM100 diet. In support of this hypothesis, an earlier study attested that Zn excess up-regulated fatty acid synthetase mRNA levels in yellow catfish, *Pelteobagrus fulvidraco* [55]. However, the effect of corn Zn excess on lipid metabolism in Nile tilapia remains largely unknown.

Fish fed on OTM-based diets have similar mineral composition throughout their bodies and vertebrae herein. The findings of Pierri et al. [50], who observed significant differences in the mineral composition of the entire body of Nile tilapia, particularly Fe, Zn, and Cu, disagree with those of this study. The types and sources of organic minerals used may have contributed to the differences found in the study. Minerals have a direct impact on the balance of macronutrients in fish metabolism [7, 8] and impact whole-body mineral retention, as previously noted in the study conducted in Nile tilapia fed with OTM-added diets in replacement of ITM [50]. Furthermore, it has been demonstrated that a severe Zn shortage in rainbow trout reduces moisture content, while increasing protein concentrations [56, 57]. Ultimately, this study evidenced that fish fed with OTM-contained diets showed higher ADC of Fe, thereby increasing its whole-body retention. Previous research reported that dietary Fe inclusion levels directly impacted the production and concentration of n-3 PUFA fatty acids in rainbow trout [58]. Excess Fe is also known to be hazardous because of its capacity to catalyze the production of free radicals and harm cellular macromolecules [59], and the effects of dietary available Fe levels on oxidative stress should be considered in future studies evaluating the OTM inclusion in Nile tilapia diets.

Plasmatic biochemical profiles are frequently used as a method to assess fish stress. As a result, the present study's findings indicate that OTM levels do not stress Nile tilapia. While AST, ALT, total protein, glucose, triglycerides, and cholesterol were not significantly impacted, there was a significant drop in ALP activity, mainly in fish fed on OTM50 to OTM75 diets. Previous research showed that increased ALP activity may be attributed to the increased Zn availability in Nile tilapia [33] to the fish, as Zn acts as a cofactor for this enzyme [53]. In aquatic animals, ALP participates in bone formation and in membrane transport activities [60]. Notably, the ALP activity of fish-fed diet OTM50 remained above the level of 40 U mg^−1^ protein observed in liver tissue of healthy tilapia, *Oreochromis* sp [61]. However, how dietary Mn and Cu levels may affect ALP activity in Nile tilapia could be further validated in future studies involving Nile tilapia.

In this study, OTM in the diet of Nile tilapia did not significantly affect intestinal morphometry. Contrary to what was observed in this investigation, previous research has found that dietary levels of OTM directly impacted the gut morphometry of Nile tilapia [50]. According to the authors, fish fed with higher levels of organic mineral intake showed lower area occupied by the fold and lamina propria. The discrepancy may be due to variations in the parameters measured and the type and levels of OTM employed. The overall fold area in the anterior region was inversely connected to the doses of minerals supplements herein. Considering this, it is feasible that increased surface area will improve mineral absorption at lower doses to match that of fish receiving greater mineral doses. Together, these results suggest that within certain limits, fish may adapt their gut morphology to optimize mineral utilization in response to its availability.

The minerals source and level of inclusion reduced the amount of Zn, Mn, and Cu in the intake and retention of minerals by fish herein, consequently affecting the extent of mineral loss. It became evident that reducing supplementation by including OTM reduced their loss by fish. Thus, we confirmed the hypothesis that dietary OTM are more available than the ITM evaluated herein. Similarly, previous studies have attested that substituting high doses of ITM for low doses of OTM did not affect Nile tilapia's growth performance [50]. To the best of the authors' knowledge, this is the first study mentioning the effects of reducing Zn, Mn, and Cu loss in Nile tilapia fed with OTM as glycinates substituting ITM. In light of environmentally sustainable fish production, the OTM used in this study can be seen as a promising nutrition tool to improve the efficiency of Zn, Mn, and Cu utilization and reduce their pollution potential. These insights have been recently emphasized in the assessment of environmentally sustainable aquaculture [28, 29]. Therefore, organic Zn, Mn, and Cu would benefit the aquaculture certification standards program and align with the sustainable principles of global fish culture.

This study indicates that fish fed on the OTM50 diet showed higher whole-body Fe content than those fed with the control diet. Previous research has shown that excess Fe is hazardous as it catalyzes the production of free radicals, which can damage cellular macromolecules [59]. When too much of an element is taken and absorbed, toxicity can result in harm that is just as severe as or worse than that brought on by deficiency [6, 7, 9, 10]. Fish and other vertebrates, therefore, integrate the many aspects of absorption, storage, and excretion to maintain a delicate equilibrium between the amounts of microminerals in the body [62]. Therefore, even in fish-fed diets with higher levels of supplementation, the saturation capacity of metals indicates the presence of a regulatory mechanism that protects tissues from excessive metal deposition and its potential harm [9]. Although a link between the concentrations of the target minerals in the diet and their body deposition was observed herein, the values discovered in the current study differ from those of earlier research [59, 62]. Although the species under consideration are the same, changes in lineage, size, food components, and microminerals sources between the studies may explain the observed variances. Overall, the complete substitution of inorganic Zn, Mn, and Cu in their sulfate forms with their corresponding 50% counterparts in organic glycinate form did not yield adverse effects on growth and feed efficiency, while concurrently reducing their loss by the fish herein. These results propose a potentially valuable strategy involving the utilization of organically chelated Zn, Mn, and Cu with glycine to enhance sustainable tilapia production.

## 5. Conclusions

From an economic standpoint, a 50% substitution of OTM did not impact the growth or feed efficiency of juvenile Nile tilapia. However, there was evidence of a limitation in the deposition of Zn, Fe, and Cu in the fish's bodies, indicating the activation of a defense mechanism against potential tissue damage caused by mineral excess. The observed lower excretion of minerals suggests that these OTM possess higher bioavailability than their inorganic counterparts, implying that Nile tilapia are absorbing and utilizing more minerals. This reduced mineral excretion has economic advantages as it diminishes the ongoing need for mineral supplementation. Furthermore, it could potentially mitigate the release of excess minerals into surrounding water bodies, thereby lowering the risk of eutrophication and ecological harm. This is crucial for maintaining the health of aquatic environments.

## Figures and Tables

**Table 1:** Ingredients and composition (g kg^−1^, on dry matter basis) of the experimental diets.

Ingredient	Diets^1^			
	ITM100	OTM100	OTM75	OTM50
Soybean meal^2^	400	400	400	400
Corn^2^	150.443	150.443	150.443	150.443
Wheat bran^2^	150	150	150	150
Rice bran^2^	80	80	80	80
Soy concentrate protein^2^	80	80	80	80
Corn gluten meal^2^	50	50	50	50
Soybean oil^2^	30	30	30	30
Dicalcium phosphate^2^	28	28	28	28
Limestone^2^	8	8	8	8
Amino acid mixture^3^	6.0	6.0	6.0	6.0
DL-methionine^4^	2.5	2.5	2.5	2.5
Mineral and vitamin mix^5^	5	5	5	5
Zinc-sulfate^4^	0.322	—	—	—
Manganese-sulfate^4^	0.020	—	—	—
Copper-sulfate^4^	0.015	—	—	—
Zinc-glycinate^6^	—	0.273	0.205	0.137
Manganese-glycinate^6^	—	0.030	0.023	0.015
Copper-glycinate^6^	—	0.016	0.012	0.008
Salt^2^	3.5	3.5	3.5	3.5
Inert (silica)^4^	—	0.038	0.122	0.196
Marker (Cr_2_O_3_)^4^	5	5	5	5
Antioxidant^7^	0.2	0.2	0.2	0.2
Antifungal^8^	1	1	1	1
Analyzed composition (g kg^−1^, dry matter)^5^				
Gross energy (MJ kg^−1^)	15.2	15.1	15.1	15.2
Crude protein	367.3	367.1	367.2	367.2
Crude fiber	46.3	46.2	46.2	46.4
Crude lipids	54.3	54.7	54.3	54.5
Calcium	11.3	11.2	11.3	11.3
Phosphorus	10.7	10.3	10.6	10.5
Iron (mg kg^−1^)	146.4	144.1	145.3	126.9

^1^ITM100: control diet was elaborated to meet the dietary requirements of Zn, Mn, and Cu from inorganic sulfate minerals (ITM100). OTM100, OTM75, and OTM50: diets were elaborated using organic Zn-glycinate, Mn-glycinate, and Cu-glycinate to provide the respective minerals at 100% (OTM100), 75% (OTM75), and 50% (OTM50) of the dietary requirements established in the control diet, respectively. ^2^Supplied by Alisul Alimentos—Supra, Maringá, PR, Brazil. ^3^Amino acid mixture (Ajinomoto Animal Nutrition Division, São Paulo, SP, Brazil), L-lysine, 3.5 g kg^−1^; L-histidine, 1 g kg^−1^; L-threonine, 1 g kg^−1^; L-tryptophan, 0.5 g kg^−1^. ^4^Sygma-Aldrich Brazil Ltda, 99.5%, São Paulo, SP, Brazil. ^5^Customized vitamin and mineral mix (composition per kilogram of diet): vitamin A (retinyl acetate), 6,000 UI; vitamin D_3_ (cholecalciferol), 1,000 UI; vitamin E (DL-*α*-tocopheryl acetate), 60 mg; vitamin K_3_ (menadione bisulfate Na), 12 mg; vitamin B_1_ (thiamine HCl), 24 mg; vitamin B_2_ (riboflavin), 24 mg; vitamin B_6_ (pyridoxine HCl), 20 mg; vitamin B_12_ (cyanocobalamin), 0.05 mg; folic acid, 6 mg; D-calcium pantothenate, 60 mg; ascorbic acid (ascorbyl polyphosphate), 240 mg; d-biotin, 0.24 mg; choline chloride, 325 mg; niacin, 120 mg; ferrous sulfate (FeSO_4_·7H_2_O), 30 mg; potassium iodide (KI), 0.4 mg, cobalt sulfate (CoSO_4_·4H_2_O), 0.25 mg; sodium selenite (Na_2_SeO^3^) = 0.1 mg. ^6^Zinc, manganese, and copper as glycinates (BASF, Corporation, Ludwigshafen, Germany). ^7^Butylhydroxytoluene (C_15_H_24_O), Sygma-Aldrich Brazil Ltda, 99.5%, São Paulo, SP, Brazil. ^8^Calcium propionate (C_6_H_10_CaO_4_), Sygma-Aldrich Brazil Ltda, 99.5%, São Paulo, SP, Brazil.

**Table 2:** Source, inclusion level, and analyzed composition (mg kg ^−1^, dry matter) of minerals in the experimental diets.

Fonte	Diet^1^			
	ITM100	OTM100	OTM75	OTM50
Inorganic source (1)				
Zn-sulfate	79.5	—	—	—
Mn-sulfate	7.0	—	—	—
Cu-sulfate	4.0	—	—	—
Organic source (2)				
Zn-glycinate	—	79.5	59.6	29.8
Mn-glycinate	—	7.0	5.3	3.5
Cu-glycinate	—	4.0	3.0	2.0
Feed ingredient (3)				
Zn	53.0	51.0	49.6	61.2
Mn	43.9	44.9	43.8	43.8
Cu	12.8	12.9	13.1	13.2
Composition (1 + 2 + 3)				
Zn	132.5	130.5	109.2	91.0
Mn	50.9	51.9	49.1	47.3
Cu	16.8	16.9	16.1	15.2

*Abbreviations*. Zn, zinc; Mn, manganese; Cu, copper. ^1^ITM100: control diet was elaborated to meet the dietary requirements of Zn, Mn, and Cu from inorganic sulfate minerals (ITM100). OTM100: OTM75, and OTM50: diets were elaborated using organic Zn-glycinate, Mn-glycinate, and Cu-glycinate to provide the respective minerals at 100% (OTM100), 75% (OTM75), and 50% (OTM50) of the dietary requirements established in the control diet, respectively.

**Table 3:** Growth performance of Nile tilapia fed on the experimental diets over an 8-week feeding trial.^1^

Parameter^2^	Diet^3^				*P*-value
	ITM100	OTM100	OTM75	OTM50	
IBW	2.9 ± 0.01	2.9 ± 0.03	2.8 ± 0.00	2.9 ± 0.02	0.203
FBW	97.2 ± 0.8	99.7 ± 2.7	94.0 ± 0.7	99.9 ± 2.7	0.163
BWG	3,276 ± 37	3,342 ± 103	3,219 ± 22	3,388 ± 102	0.408
RFI	2.9 ± 0.00	2.9 ± 0.00	2.9 ± 0.00	2.9 ± 0.00	0.416
FER	1.09 ± 0.00	1.08 ± 0.01	1.09 ± 0.00	1.10 ± 0.01	0.480
PRE	44.7 ± 0.7	45.0 ± 0.4	44.3 ± 0.4	44.6 ± 0.7	0.557
ERE	36.7 ± 0.2	36.7 ± 0.1	36.8 ± 0.4	37.6 ± 0.4	0.158
VSI	10.3 ± 0.4	10.6 ± 0.2	10.5 ± 0.2	9.6 ± 0.8	0.395
HSI	3.1 ± 0.1	3.5 ± 0.2	3.3 ± 0.1	3.1 ± 0.1	0.351
VFR	2.3 ± 0.1	1.8 ± 0.1	2.2 ± 0.2	2.2 ± 0.2	0.140
SUR	100.0 ± 0.0	99.1 ± 0.9	100.0 ± 0.0	100.0 ± 0.0	0.426

^1^Data are means ± standard error of the mean of four replicates (27 fish per replicate). ^2^IBW, initial body weight (g); FBW, final body weight (g); BWG, body weight gain (%); RFI, relative feed intake (%body weight day^−1^); FER, feed efficiency ratio; PRE, protein retention efficiency (%); ERE, energy retention efficiency (%); VSI, viscerosomatic index (%); HIS, hepatosomatic index (%); VFR, visceral fat ratio (%); SUR, survival (%). ^3^ITM100: control diet was elaborated to meet the dietary requirements of Zn, Mn, and Cu from inorganic sulfate minerals (ITM100). OTM100, OTM75, and OTM50: diets were elaborated using organic Zn-glycinate, Mn-glycinate, and Cu-glycinate to provide the respective minerals at 100% (OTM100), 75% (OTM75), and 50% (OTM50) of the dietary requirements established in the control diet, respectively.

**Table 4:** Whole-body composition (g kg^−1^, wet basis) of Nile tilapia fed the experimental diets over an 8-week feeding trial.^1^

Parameter^2^	Diet^3^				*P*-value
	ITM100	OTM100	OTM75	OTM50	
Whole-body					
MO	722.0 ± 0.8	723.4 ± 2.1	727.0 ± 7.2	721.3 ± 1.0	0.727
CP	142.3 ± 1.1	140.6 ± 1.3	136.9 ± 3.0	138.8 ± 2.9	0.401
CL	77.3 ± 1.5^a^	64.4 ± 2.5^b^	74.3 ± 2.5^a^	77.8 ± 0.7^a^	<0.001
MM	28.9 ± 0.71^b^	33.4 ± 0.7^a^	33.6 ± 0.5^a^	31.9 ± 1.4^ab^	0.001

^1^Data are means ± standard error of the mean of four replicates (27 fish per replicate). ^2^ MO, moisture; CP, crude protein; CL, crude lipids; MM, mineral matter. ^3^ITM100: control diet was elaborated to meet the dietary requirements of Zn, Mn, and Cu from inorganic sulfate minerals (ITM100). OTM100, OTM75, and OTM50: diets were elaborated using organic Zn-glycinate, Mn-glycinate, and Cu-glycinate to provide the respective minerals at 100% (OTM100), 75% (OTM75), and 50% (OTM50) of the dietary requirements established in the control diet, respectively. ^a,b^Means in the same line with different superscripts are significantly different by Tukey's test (*P* ≤ 0.05).

**Table 5:** Whole-body and vertebrae minerals composition (g kg^−1^, wet basis) of Nile tilapia fed on the experimental diets over 8-week feeding trial.^1^

Parameter^2^	Diet^3^				*P*-value
	ITM100	OTM100	OTM75	OTM50	
Whole-body					
Ca	2.5 ± 0.0	2.6 ± 0.0	2.6 ± 0.1	2.6 ± 0.0	0.231
P	2.0 ± 0.0	1.8 ± 0.1	1.9 ± 0.1	1.9 ± 0.0	0.383
Zn	25.5 ± 1.0	26.3 ± 1.0	25.5 ± 0.8	26.1 ± 1.0	0.928
Mn	2.8 ± 0.1	2.8 ± 0.2	2.6 ± 0.2	2.9 ± 0.1	0.664
Cu	1.6 ± 0.1	1.7 ± 0.1	1.5 ± 0.0	1.5 ± 0.0	0.108
Fe	72.4 ± 2.4	76.3 ± 3.2	71.5 ± 1.7	78.7 ± 1.6	0.125
Vertebrae					
Ca	114.2 ± 2.5	118.1 ± 0.8	118.3 ± 1.2	116.2 ± 1.1	0.256
P	135.2 ± 11.8	127.4 ± 1.5	167.8 ± 6.8	131.6 ± 16.6	0.068
Zn	42.0 ± 1.4	42.9 ± 1.8	47.6 ± 2.9	40.6 ± 2.7	0.200
Mn	0.9 ± 0.0	1.0 ± 0.1	1.0 ± 0.0	1.0 ± 0.0	0.354
Cu	1.5 ± 0.0	1.6 ± 0.1	1.4 ± 0.0	1.4 ± 0.1	0.138
Fe	62.9 ± 3.9	64.7 ± 3.0	65.3 ± 4.9	72.8 ± 5.9	0.125

^1^Data are means ± standard error of the mean of four replicates (27 fish per replicate). ^2^Ca, calcium; P, phosphorus; Zn, zinc; Mn, manganese; Cu, copper; Fe, iron. ^3^ITM100: control diet was elaborated to meet the dietary requirements of Zn, Mn, and Cu from inorganic sulfate minerals (ITM100). OTM100, OTM75, and OTM50: diets were elaborated using organic Zn-glycinate, Mn-glycinate, and Cu-glycinate to provide the respective minerals at 100% (OTM100), 75% (OTM75), and 50% (OTM50) of the dietary requirements established in the control diet, respectively.

**Table 6:** Whole-body minerals retention (%) of Nile tilapia fed on the experimental diets over 8-week feeding trial.^1^

Parameter^2^	Diet^3^				*P*-value
	ITM100	OTM100	OTM75	OTM50	
Ca	24.4 ± 0.6^b^	26.9 ± 0.2^a^	26.9 ± 0.7^a^	26.6 ± 0.3^a^	0.011
P	21.8 ± 0.2	20.3 ± 0.8	20.7 ± 1.7	20.7 ± 0.3	0.694
Zn	20.6 ± 0.9^c^	21.3 ± 0.9^bc^	25.0 ± 0.7^b^	30.9 ± 1.0^a^	<0.001
Mn	15.0 ± 0.7	15.5 ± 1.1	16.4 ± 0.3	17.8 ± 0.3	0.075
Cu	25.4 ± 0.6	29.4 ± 1.0	25.2 ± 0.7	28.9 ± 1.1	0.138
Fe	50.9 ± 2.1^b^	56.5 ± 3.2^b^	60.6 ± 2.6^ab^	72.8 ± 4.3^a^	0.002

^1^Data are means ± standard error of the mean of four replicates (27 fish per replicate). ^2^Ca, calcium; P, phosphorus; Zn, zinc; Mn, manganese; Cu, copper; Fe, iron. ^3^ITM100: control diet was elaborated to meet the dietary requirements of Zn, Mn, and Cu from inorganic sulfate minerals (ITM100). OTM100, OTM75, and OTM50: diets were elaborated using organic Zn-glycinate, Mn-glycinate, and Cu-glycinate to provide the respective minerals at 100% (OTM100), 75% (OTM75), and 50% (OTM50) of the dietary requirements established in the control diet, respectively. ^a–c^Means in the same line with different superscripts are significantly different by Tukey's test (*P* ≤ 0.05).

**Table 7:** Plasmatic biochemical values of Nile tilapia fed on the experimental diets over 8-week feeding trial.^1^

Parameter^2^	Diet^3^				*P*-value
	ITM100	OTM100	OTM75	OTM50	
ALP	68.9 ± 8.0^ab^	84.7 ± .0.9^a^	62.0 ± 6.9^ab^	40.2 ± 8.7^b^	0.005
AST	32.7 ± 6.4	51.5 ± 9.8	36.2 ± 5.6	31.9 ± 5.0	0.212
ALT	17.5 ± 0.1	39.3 ± 4.4	31.1 ± 8.5	26.2 ± 5.1	0.080
TP	1.8 ± 0.1	1.9 ± 0.1	1.9 ± 0.2	1.8 ± 0.1	0.929
GLU	65.2 ± 3.7	78.9 ± 5.0	77.4 ± 4.5	64.5 ± 3.4	0.056
TGC	295 ± 321	345.9 ± 44.1	257.4 ± 25.7	397.2 ± 64.7	0.184
CHO	98.0 ± 5.9	106.2 ± 10.7	99.9 ± 3.2	95.9 ± 3.9	0.720

^1^Data are means ± standard error of the mean of four replicates (27 fish per replicate). ^2^ALP, alkaline phosphatase (U l^−1^); AST, aspartate aminotransferase (U l^−1^); ALT, alanine aminotransferase (U l^−1^); TP, total protein (g dl^−1^); GLU, glucose (mg dL^−1^); TGC, triglycerides (mg dL^−1^); CHO, cholesterol (mg dL^−1^). ^3^ITM100: control diet was elaborated to meet the dietary requirements of Zn, Mn, and Cu from inorganic sulfate minerals (ITM100). OTM100, OTM75, and OTM50: diets were elaborated using organic Zn-glycinate, Mn-glycinate, and Cu-glycinate to provide the respective minerals at 100% (OTM100), 75% (OTM75), and 50% (OTM50) of the dietary requirements established in the control diet, respectively. ^a,b^Means in the same line with different superscripts are significantly different by Tukey's test (*P* ≤ 0.05).

**Table 8:** Gut histomorphometry (*µ*m) of juvenile Nile tilapia fed on the experimental diets over 8-week feeding trial.^1^

Parameter	Diet^2^				*P*-value
	ITM100	OTM100	OTM75	OTM50	
Villus height (*µ*m)	47.0 ± 1.9	54.9 ± 2.1	52.4 ± 3.9	49.5 ± 1.9	0.249
Villus width (*µ*m)	18.4 ± 0.6	19.2 ± 11.1	21.2 ± 1.3	19.6 ± 0.8	0.537
Villus height: villus width	2.6 ± 0.1	2.9 ± 0.1	2.5 ± 0.2	2.5 ± 0.0	0.167
Area of absorption (*µ*m^2^)	864.3 ± 47.0	1,056.7 ± 87.0	1,138.6 ± 107.3	977.3 ± 75.6	0.237

^1^Data are means ± standard error of the mean of four replicates (27 fish per replicate). ^2^ITM100: control diet was elaborated to meet the dietary requirements of Zn, Mn, and Cu from inorganic sulfate minerals (ITM100). OTM100, OTM75, and OTM50: diets were elaborated using organic Zn-glycinate, Mn-glycinate, and Cu-glycinate to provide the respective minerals at 100% (OTM100), 75% (OTM75), and 50% (OTM50) of the dietary requirements established in the control diet, respectively.

**Table 9:** Apparent digestibility coefficients of the experimental diets in Nile tilapia fed the experimental diets over an 8-week feeding trial.^1^

Parameter (%)^2^	Diet^3^				*P*-value
	ITM100	OTM100	OTM75	OTM50	
GE	73.6 ± 0.1^d^	80.2 ± 0.1^a^	78.2 ± 0.2^b^	74.3 ± 0.2^c^	<0.001
CP	91.7 ± 0.3^b^	93.8 ± 0.1^a^	93.4 ± 0.0^a^	92.1 ± 0.1^b^	<0.001
Ca	61.1 ± 1.5	62.4 ± 0.7	61.5 ± 1.0	60.3 ± 0.5	0.333
P	60.3 ± 0.1	61.8 ± 0.1	60.7 ± 0.2	61.4 ± 1.7	0.618
Zn	59.4 ± 1.2^ab^	56.6 ± 0.7^b^	59.9 ± 1.7^ab^	61.2 ± 1.8^a^	0.023
Mn	58.3 ± 1.7	60.5 ± 1.2	54.4 ± 2.1	55.0 ± 2.0	0.099
Cu	67.6 ± 6.3	62.4 ± 3.4	66.7 ± 8.4	65.5 ± 9.1	0.201
Fe	60.6 ± 5.4^b^	76.7 ± 2.3^ab^	71.6 ± 1.5^ab^	80.6 ± 6.5^a^	0.038

^1^Data are means ± standard error of the mean of four replicates (27 fish per replicate). ^2^GE, gross energy; Ca, calcium; P, phosphorus, Zn, zinc; Mn, manganese; Cu, copper; Fe, iron). ^3^ITM100: control diet was elaborated to meet the dietary requirements of Zn, Mn, and Cu from inorganic sulfate minerals (ITM100). OTM100, OTM75, and OTM50: diets were elaborated using organic Zn-glycinate, Mn-glycinate, and Cu-glycinate to provide the respective minerals at 100% (OTM100), 75% (OTM75), and 50% (OTM50) of the dietary requirements established in the control diet, respectively.^a–c^Means in the same line with different superscripts are significantly different by Tukey's test (*P* ≤ 0.05).

**Table 10:** Minerals balance (mg kg^−1^, body weight gain of fish) of Nile tilapia fed with the experimental diets over 8-week feeding trial.^1^

Parameter^2^	Diet^3^				*P*-value
	ITM100	OTM100	OTM75	OTM50	
Zn					
Intake	120.2 ± 0.3^a^	121.2 ± 1.4^a^	100.3 ± 0.3^b^	83.0 ± 0.3^c^	<0.001
Retained	24.8 ± 1.2	25.9 ± 1.1	25.1 ± 0.8	25.6 ± 1.0	0.870
Loss	95.4 ± 0.9^a^	95.3 ± 1.6^a^	75.2 ± 0.6^b^	57.4 ± 0.5^c^	<0.001
Mn					
Intake	46.9 ± 0.1^a^	47.3 ± 0.5^a^	45.1 ± 0.1^b^	43.0 ± 0.4^c^	<0.001
Retained	7.2 ± 0.4	7.3 ± 0.4	6.4 ± 0.6	7.6 ± 0.2	0.298
Loss	39.7 ± 0.3^a^	39.9 ± 0.9^a^	38.6 ± 0.6^b^	35.3 ± 0.2^c^	<0.001
Cu					
Intake	15.5 ± 0.0^a^	15.7 ± 0.2^a^	14.9 ± 0.0^b^	13.8 ± 0.1^c^	<0.001
Retained	4.2 ± 0.2^b^	4.6 ± 0.2^a^	4.0 ± 0.2^b^	4.0 ± 0.1^b^	<0.001
Loss	11.4 ± 0.2^a^	11.1 ± 0.1^a^	11.1 ± 0.1^a^	9.8 ± 0.2^b^	<0.001

^1^Data are means ± standard error of the mean of four replicates (27 fish per replicate aquaria). ^2^Zn, zinc; Mn, manganese; Cu, copper. ^3^ITM100: control diet was elaborated to meet the dietary requirements of Zn, Mn, and Cu from inorganic sulfate minerals (ITM100). OTM100, OTM75, and OTM50: diets were elaborated using organic Zn-glycinate, Mn-glycinate, and Cu-glycinate to provide the respective minerals at 100% (OTM100), 75% (OTM75), and 50% (OTM50) of the dietary requirements established in the control diet, respectively. ^a–c^Means in the same line with different superscripts are significantly different by Tukey's test (*P* ≤ 0.05).

## Data Availability

Data will be made available upon request.
